# Characterization of extracellular polymeric substances (EPS) from periphyton using liquid chromatography-organic carbon detection–organic nitrogen detection (LC-OCD-OND)

**DOI:** 10.1007/s11356-012-1228-y

**Published:** 2012-10-12

**Authors:** Theodora J. Stewart, Jacqueline Traber, Alexandra Kroll, Renata Behra, Laura Sigg

**Affiliations:** 1 Eawag, Swiss Federal Institute of Aquatic Science and Technology, 8600 Dübendorf, Switzerland; 2 ETH, Institute of Biogeochemistry and Pollutant Dynamics IBP, 8092 Zurich, Switzerland

**Keywords:** Periphyton, Biofilm, EPS, Characterization, LC-OCD-OND, Polysaccharide, Protein

## Abstract

A protocol was developed to extract, fractionate, and quantitatively analyze periphyton extracellular polymeric substances (EPS), which obtains both information on the molecular weight (*M*_r_) distribution and protein and polysaccharide content. The EPS were extracted from freshwater periphyton between July and December 2011. Organic carbon (OC) compounds from different EPS extracts were analyzed using liquid chromatography-organic carbon detection–organic nitrogen detection (LC-OCD-OND), and total protein and polysaccharide content were quantified. Four distinct OC fractions, on the basis of *M*_r_, were identified in all extracts, corresponding to high *M*_r_ biopolymers (≥80–4 kDa), degradation products of humic substances (*M*_r_ not available), low *M*_r_ acids (10–0.7 kDa), and small amphiphilic/neutral compounds (3–0.5 kDa). Low C/N ratios (4.3 ± 0.8) were calculated for the biopolymer fractions, which represented 16–38 % of the measured dissolved organic carbon (DOC), indicating a significant presence of high *M*_r_ proteins in the EPS. Protein and polysaccharide represented the two major components of EPS and, when combined, accounted for the measured DOC in extracts. Differences in specific OC fractions of EPS extracts over the course of the study could be quantified using this method. This study suggests that LC-OCD-OND is a new valuable tool in EPS characterization of periphyton.

**Electronic supplementary material:**

The online version of this article (doi:10.1007/s11356-012-1228-y) contains supplementary material, which is available to authorized users.

## Introduction

Phototrophic biofilms, also commonly referred to as periphyton, are communities of hetero- and autotrophic organisms held together by a network of extracellular polymeric substances (EPS). The excreted EPS, typically composed of polysaccharides, proteins, glycoproteins, glycolipids, nucleic acids, and amphiphilic compounds (Cogan and Keener 2004; Neu and Lawrence 2009; Stoodley et al. 2002), provide both biofilm structure and a nutrient source for periphytic organisms, but also may serve as a protective barrier against toxic compounds, such as nonessential trace metals.

Methods of EPS characterization have largely relied on techniques for quantification of total proteins and polysaccharides in EPS, using assays, such as the Bradford and Lowry, and the DuBois methods (Bradford 1976; DuBois et al. 1956). Despite the relative ease of standard assays, these methods only provide information on total concentrations in a sample. More detailed characterization techniques, such as HPLC, have been used to determine the monosaccharide composition in both bacterial and phototrophic biofilms (Celik et al. 2008; Congestri 2006; Meisen et al. 2008), coupled mass spectrometry (MS) techniques, such as LC-MS/MS, have been used to identify specific proteins in bacterial biofilms (Cao et al. 2011), and confocal scanning laser microscopy has been used to study the exopolysaccharide composition of biofilms using fluorescently labeled lectins (Zippel and Neu 2011). These methods have the advantage of targeting and identifying specific types of extracellular compounds. However, what is needed is a comprehensive and quantitative approach, able to simultaneously fractionate and characterize EPS.

Liquid chromatography-organic carbon detection–organic nitrogen detection (LC-OCD-OND) is a technique based on size-exclusion chromatography (SEC), which is able to provide quantitative information regarding organic carbon (OC) compounds, such as polysaccharides and proteins found in EPS. Although SEC has been previously used for the separation of compounds in bacterial EPS (Ras et al. 2011; Simon et al. 2009; Villain et al. 2010), LC-OCD-OND provides additional information by the online coupling of SEC to OC, organic nitrogen (ON), and ultraviolet (UV) detectors. The total (TOC) and dissolved organic carbon (DOC) are measured, and DOC is further separated and quantified in six separate fractions: biopolymers (high *M*_r_ polysaccharides and proteins), humic substances (HS), building blocks of HS, low *M*_r_ acids, and amphiphilic/neutral compounds (alcohols, aldehydes, ketones, and amino acids). This technique has been used in the drinking and groundwater monitoring sectors, and there is a collection of work that has studied different fractions of EPS from bacterial biofilms, with respect to assessing membrane fouling (Al-Halbouni et al. 2008; Meng et al. 2009; Zheng et al. 2009). However, LC-OCD-OND has never been used as a tool to characterize EPS from periphyton.

The aim of this work was twofold: to develop an extraction protocol for EPS from periphyton suitable for metal analysis and to obtain *M*_r_ and protein and polysaccharide content of different OC fractions of EPS using LC-OCD-OND. The EPS was extracted from biofilms, colonized between July and December 2011. Chromatograms from this 6-month period were compared to determine if differences in EPS composition could be observed and quantified using this technique. Results were compared with total polysaccharide and protein quantification. The application of LC-OCD-OND for the characterization of periphyton EPS is assessed.

## Materials and methods

### Periphyton colonization

All periphyton was colonized on glass microscope slides, previously acid soaked in 0.03 M HNO_3_ and washed with nanopure water (Ω 18, Milli-Q), which were placed in Plexiglas flow-through channels with continuous pumping of natural stream water from the Chriesbach (Dübendorf, Switzerland) (Navarro et al. 2008). Each channel contained four rows of eight paired glass slides (76 × 26 mm, Thermo Scientific), amounting to a total of 64 slides per channel. Larger-sized sediment was removed from the water before entering colonization channels by using a sediment trap (51 × 70 × 260 cm) with an average residence time of 20 min. Flow rate was maintained at approximately 1 cm/s and monitored with a Schildknecht MiniAir2 flow meter. Illumination was provided by BioSun fluorescent tubes (MLT Moderne Licht-Technik AG, ML-T8 36W/965/G13B), mimicking the natural sunlight spectrum. Light/dark cycles, each 12 h, were maintained with electronically controlled timers (Demelectric AG). Slides were transferred to a plastic box containing stream water, so that drying of the biofilms was avoided during transport, prior to extraction.

### Periphyton EPS extraction

Glass slides were taken from flow-through channels after 25 days of colonization. Biomass was gently scraped off with a clean glass slide into a 100-mL muffled glass beaker placed on ice containing the extraction solution composed of NaNO_3_ (10 mM, pH 7.4) and a 1-μg/mL protease inhibitor cocktail, with equal amounts of Aprotinin, Leupeptin, and Pepstatin A (AppliChem AG). The 10-mM NaNO_3_ was chosen, as it is a chemically inert salt with an ionic strength similar to the stream water used to colonize the periphyton. A volume of 60 mL was used for the extraction of 32 slides. The biofilm slurry was resuspended in the solution by gentle pipetting and then further resuspended using a water sonication bath (45 kHz 60 W, VWR Ultrasonic Cleaner) for 30 s. Fine sediment and larger biomass was allowed to settle, and the solution was removed and centrifuged at 1,880×*g* for 10 min. Biomass was resuspended a second time in fresh solution and treated as described above. In a separate series of extractions, NaHCO_3_ (2 mM, pH 7.6) was used as an extraction solution.

To determine if agitation of the solution over time yielded higher EPS extraction efficiencies, two different physical methods were compared, referred to as shaking and stirring methods. A biofilm slurry was obtained according to the above protocol and a control sample, representing *t* = 0, was taken before the remaining slurry was separated into separate aliquots. Half of the aliquots were placed on a shaker at 90 rpm, whereas the remaining aliquots were stirred at 300 rpm. Samples were taken after 30, 60, 90, and 120 min from each type of extraction method and centrifuged at 1,880×*g* for 10 min. The resulting biomass pellet was lyophilized overnight, and dry weight measurements were taken. All supernatants were filtered through 0.22 μm PES Millipore filters, which were previously flushed with 1 L of nanopure water (Ω 18, Milli-Q) to prevent OC contamination of the sample. Filtered samples were stored in muffled 100-mL glass Schott flasks at 4 °C and treated with a final concentration of 0.02 % (*w*/*v*) NaN_3_ to prevent bacterial growth and subsequent degradation of EPS.

### Glucose-6-phosphate dehydrogenase assay

Activity of glucose-6-phosphate dehydrogenase (G6P-DH), an intracellular enzyme, was measured in extracts according to Esposito et al., to determine the degree of cell lysis resulting from the extraction technique used in this study (Esposito et al. 2006). At each step of the extraction (i.e., scraping of biofilms from slides, sonication, centrifugation, and filtration), samples were taken in triplicate and transferred to a 96-well plate. Upon addition of 180 μL of reaction mixture (50 mM Tris Base, 0.15 mM NADP, 10 mM MgCl_2_, and 3 mM glucose-6-phosphate), absorption of NADPH (formed during the conversion of glucose-6-phosphate) was measured at 340 nm at 30 °C over 30 min. A standard curve was generated using G6P-DH in both water and in the EPS extract to verify that the EPS did not interfere with the detection of G6P-DH activity. The limit of detection in both cases was 0.00125 U/mL (U = the amount of enzyme that reduces 1.0 μmol NADP/min at 30 °C, pH 7.8). To measure total activity of the biomass, cell lysis was induced by combining 1 mL of the biomass suspension with the same volume of extraction solution containing 0.1 % SDS. The sample was sonicated for 15 min, centrifuged, and the supernatant was used as a representation of total G6P-DH activity. A known amount of G6P-DH was also treated as described above to verify that the procedure did not inhibit G6P-DH activity.

### LC-OCD-OND characterization of extracted EPS

To characterize the OC compounds found in the extracted EPS, LC-OCD-OND was used. Samples were diluted (1:50) with nanopure water (Ω 18, Milli-Q) in muffled assimilable organic carbon-free 20-mL glass vials. Compounds were separated using a size exclusion column (250 × 20 mm, Toyopearl TSK HW-50S) able to separate both polysaccharides (0.1–18 kDa) and proteins (0.5–80 kDa for globular proteins), as reported by the manufacturer. A handling control, in which the extraction solution was treated like the periphyton EPS extract, was performed to assess the carbon contamination associated with the extraction protocol. In all cases, OC coming from the handling control accounted for less than 2 % of DOC in extracts and, therefore, was negligible for the quantification of OC compounds in EPS. Phosphate buffer (24 mM, pH 6.6) was used as the mobile phase and phosphoric acid solution (60 mM, pH 1.2) was used as an acidification solution to aid in the removal of inorganic carbon prior to analysis. The limit of quantification was 10 μg/L for both OC and ON.

The TOC and DOC, as well as specific OC compounds, were identified and quantified using FIFFIKUS, a software quantification method (DOC-Labor Dr. Huber, Germany). Distinguishable fractions can include mineral colloids, polysaccharides, HS, building blocks of HS, low *M*_r_ acids, and amphiphilic/neutral compounds. The software uses information obtained from the isolation of polysaccharides, and other fractions from EPS, and their subsequent measurement using ion chromatography and amperometric detection for sugars.

### LC-OCD-OND calibration

Protein and polysaccharide standards were measured to create a calibration curve for the *M*_r_ determination of peaks from extracts. BSA (66.5 kDa), ovalbumin (44 kDa), Carbonic Anhydrase (29 kDa), Ribonuclease A (13.7 kDa), Aprotinin (6.5 kDa), and Pepstatin A (0.686 kDa) were used as protein standards. Thyroglobulin (669 kDa) was used to determine the void volume of the column and for the calculation of retention factors. Protein standards were measured both individually and in mixtures in phosphate buffer, NaNO_3_, NaHCO_3_, and spiked in EPS extracts so as to rule out matrix effects on elution times. As no significant effects were observed, the standard calibration curve in phosphate buffer was used for *M*_r_ calculations (Online resource 1 in the Electronic supplementary material (ESM)). Mixtures of protein standards (Thyroglobulin (669 kDa), BSA (66.5 kDa), Ovalbumin (44 kDa), and Pepstatin A (0.686 kDa)), corresponding to retention times of peaks seen in EPS extracts, were analyzed (Online resource 2 in the ESM) to determine the resolution of *M*_r_ separation in these mixtures. Polyethylene glycol (PEG) standards ranging from 0.106 to 21.030 kDa were used as polysaccharide standards and measured in nanopure water (Ω 18, Milli-Q), NaNO_3_, NaHCO_3_, and spiked in EPS extracts. The standard calibration curve in nanopure water was used for *M*_r_ calculations.

### Protein and polysaccharide quantification

The Bradford assay was conducted on the whole extract to quantify total protein (Bradford 1976). BSA was used for standard calibration, and extracts were diluted with nanopure water to fall within the calibration range. Bradford dye reagent (Bio-Rad) was used for the analysis. Absorbance was measured at 595 nm, and samples were measured in triplicate. Protein was converted from micrograms of protein to milligrams of carbon, assuming an average carbon content of 0.53 g C/g protein (Rouwenhorst et al. 1991).

The phenol-sulfuric acid method (DuBois method) was used on the whole extract to determine total polysaccharide content (DuBois et al. 1956). Glucose was used as a calibration standard. Equal volumes of sample or glucose standard were mixed with phenol (500 μL, 2 % (*w*/*v*), Fluka) in glass vials and then 2.5 mL of concentrated sulfuric acid (95–97 %, Sigma-Aldrich) was added, and the solution was mixed via gentle vortex. After allowing the sample to cool for 30 min, absorbance of solutions was measured at 490 nm. Polysaccharide was converted from micrograms of polysaccharide to milligrams of carbon, assuming 0.4 g C/g polysaccharide (Rouwenhorst et al. 1991).

## Results

### Extraction method

**Fig. 1:**
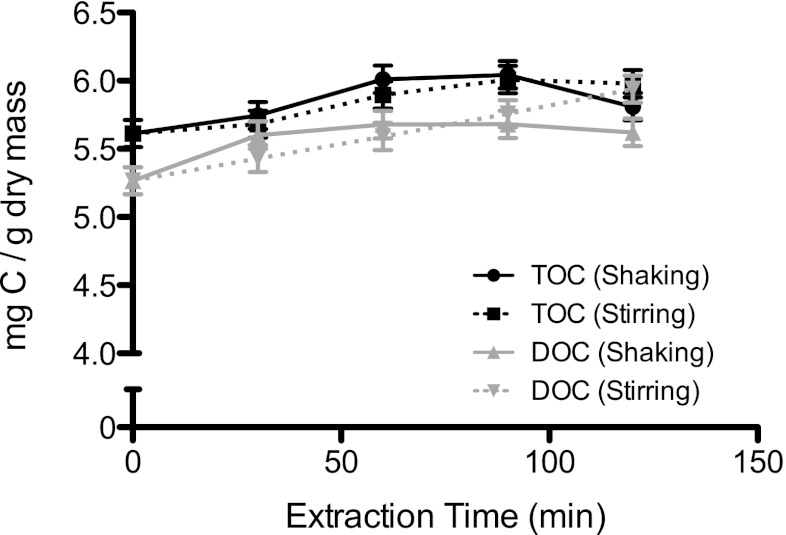
OC extraction efficiency of shaking and stirring extraction methods. Biomass slurry from harvested biofilms was split into separate aliquots, and EPS was extracted either with shaking (*solid line*) or stirring (*dashed line*) methods over 120 min in triplicate. Samples were taken at different time points for analysis with LC-OCD-OND. *Error bars* generated from standard deviation of triplicates

The EPS extraction efficiencies of the shaking and stir methods were evaluated with respect to TOC and DOC. The amount of extracted TOC and DOC are shown in Fig. 1 as a function of extraction time. No significant differences were observed between these two methods over the course of 120 min nor were differences observed over time within a single type of extraction technique. No differences were observed between NaNO_3_ and NaHCO_3_ as extraction solutions (Online resource 3 in the ESM). Results from the G6P-DH assay indicated no detectable G6P-DH activity during any step of the EPS extraction, as measurements were all below the limit of detection of the assay (Online resource 4 in the ESM).

### EPS characterization

**Fig. 2:**
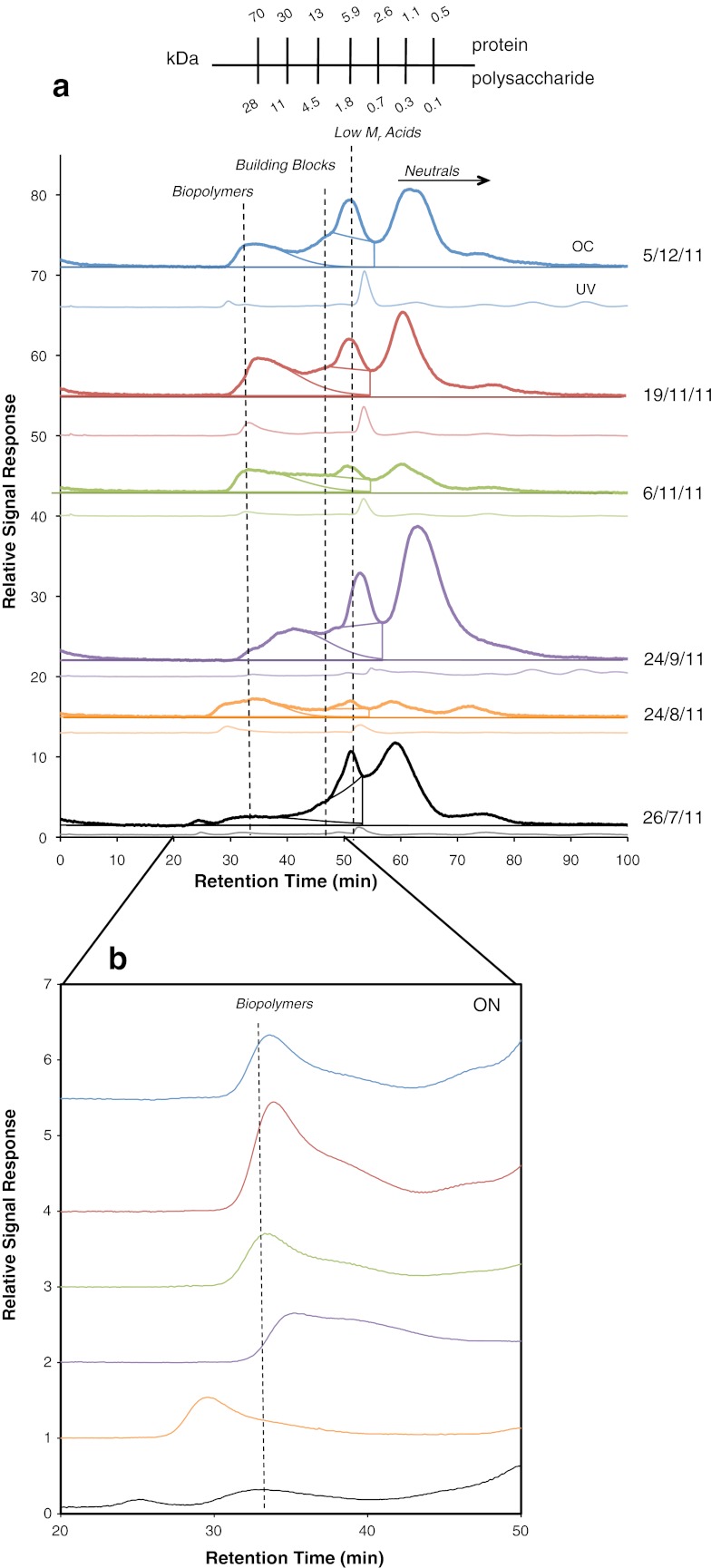
LC-OCD-OND chromatograms of periphyton EPS extracts taken between July and December 2011 from 25-day-old biofilms. **a** For each sample set, upper chromatograms correspond to OC signal and lower to UV absorbance detected at 254 nm. OC and UV chromatograms have different scaling and are not comparable. *Integration lines* are shown within OC chromatograms. *M*_r_ for protein and polysaccharide equivalents generated from calibration curve. **b** ON signals correspond to biopolymer fractions

**Fig. 3:**
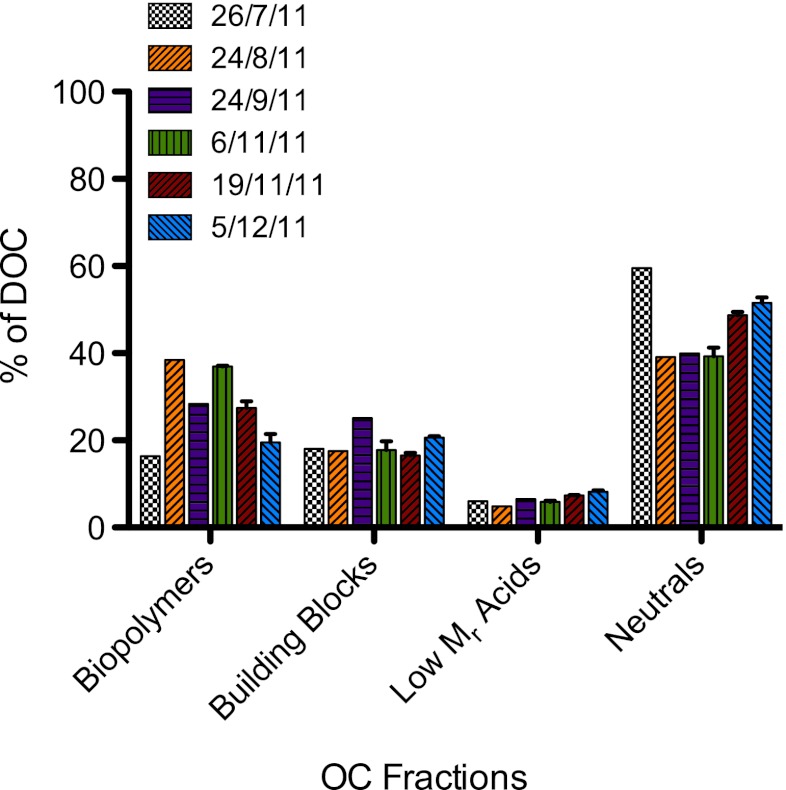
Quantification of OC fractions from periphyton EPS samples as determined by LC-OCD-OND using the software integration program FIFFIKUS (DOC-LABOR, Germany). Values expressed as percent of chromatographable DOC. Standard deviation bars were generated from replicate extractions performed on the same set of biofilms

In all LC-OCD-OND chromatograms (Fig. 2a), four distinct fractions were observed corresponding to biopolymers, building blocks of HS, low *M*_r_ acids, and neutral/amphiphilic compounds. No HS were present in any EPS extracts measured, as HS typically elute at approximately 45 min and would display a distinct signal in the UV spectra (Huber et al. 2011). However, UV absorbance (254 nm) was observed for the biopolymer and low *M*_r_ acid fractions in all extracts (Online resource 5 in the ESM). An ON signal was measured for all biopolymer fractions (Fig. 2b) but could not be assessed for the other fractions as the nitrogen signal from NaNO_3_, which was detected at the same retention time as the remaining fractions, was too large. Areas of individual fractions were integrated as shown in Fig. 2a, quantified, and are presented as a percentage of chromatographable DOC (Fig. 3).

As seen in Fig. 3, the biopolymer fraction represented 16–38 % of measured DOC, whereas the building block, low *M*_r_ acid, and neutral fractions corresponded to 16–25, 5–8, and 40–60 % DOC, respectively. Extract taken in July contained the least amount of biopolymers (16 % DOC) relative to the other OC fractions, whereas extracts from August and the beginning of November (6 November 2011) contained the largest amounts (38 and 37 % DOC, respectively). A decreasing trend was observed between November and December. From the quantification of OC and ON in the biopolymer fractions, an average C/N ratio of 4.3 ± 0.8 was calculated. The amount of building blocks varied less over the course of study, with the greatest amount found in the September extract. The low *M*_r_ acids were consistent throughout all extracts. The neutral/amphiphilic fraction corresponded to the greatest percentage of measured DOC in the EPS extracts. Similar amounts were quantified in extracts from August through early November (37–40 % DOC) and greater amounts were measured in extracts from July (60 % DOC), November (19 November 2011; 49 % DOC) and December (53 % DOC) (Fig. 3).

Calculated ranges of *M*_r_ for each fraction, using protein and polysaccharide-generated standard curves, are shown in Table 1. The *M*_r_ range of proteins in the biopolymer fraction was 12 to ≥80 kDa, whereas polysaccharides fell between 4 to ≥20 kDa. The low *M*_r_ acid fraction corresponded to a peptide range between 2 and 10 and 0.7 and 3 kDa for polysaccharides. Neutral/amphiphilic compounds corresponded to 0.5–3 kDa. The *M*_r_ of the building blocks fraction was not calculated because there were no distinct peaks corresponding to this fraction for which a *M*_r_ range could be calculated.

**Table 1:** *M*_r_ distribution of EPS fractions identified with LC-OCD-OND

Sample	Biopolymers		Low *M*_r_ acids		Neutrals	
	*M*_r_ (kDa)					
	Protein	Polysaccharide	Peptide	Polysaccharide	Peptide	Polysaccharide
5 December 2011	≥80–25	≥20–9	9–3	3–0.7	3 to ≤0.5	0.7 to ≤0.5
19 November 2011	≥80–19	≥20–7	9–3	3–0.7	3 to ≤0.5	0.7 to ≤0.5
6 November 2011	≥80–25	≥20–9	8–3	3–0.7	3 to ≤0.5	0.7 to ≤0.5
24 September 2011	≥80–12	≥20–4	7–2	2–0.7	2 to ≤0.5	0.7 to ≤0.5
24 August 2011	≥80–14	≥20–4	7–3	2–0.8	2 to ≤0.5	0.8 to ≤0.5
26 July 2011	≥80–42	≥20–15	10–3	3–0.9	3 to ≤0.5	0.9 to ≤0.5

Both protein and polysaccharide equivalent *M*_r_ ranges calculated for each sample from calibrations generated from globular protein standards and PEG standards and using min and max elution times for each fraction (Online resource 1 in the ESM)

Differences between EPS extracts obtained in replicate, taken from the same set of biofilms harvested from one channel, was less than 20 % for biopolymers and building blocks and less than 10 % for low *M*_r_ acid and neutral/amphiphilic fractions (Online resource 6 in the ESM). Variability between replicate measurements of the same sample using LC-OCD-OND was assessed by the manufacturer and reported the half confidence interval to be less than 10 % (12.4 % for the neutrals fraction) (DOC-Labor Dr. Huber, Germany).

### Total polysaccharide and protein quantification

**Fig. 4:**
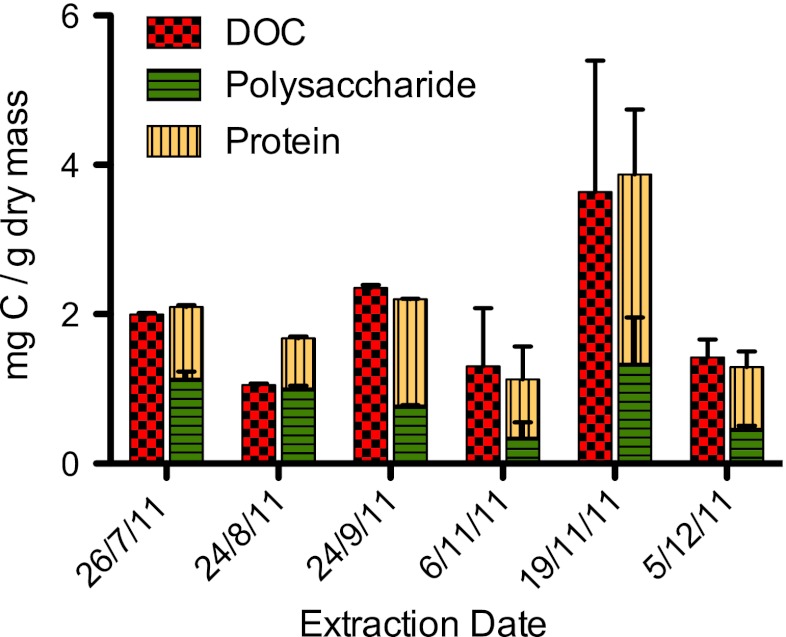
Comparison of DOC to total protein and polysaccharide from periphyton EPS extracts. Total protein and polysaccharide measured was converted to milligrams of carbon and normalized to lyophilized dry biomass to compare with measured DOC. Standard deviation bars were generated from triplicate measurements

**Fig. 5:**
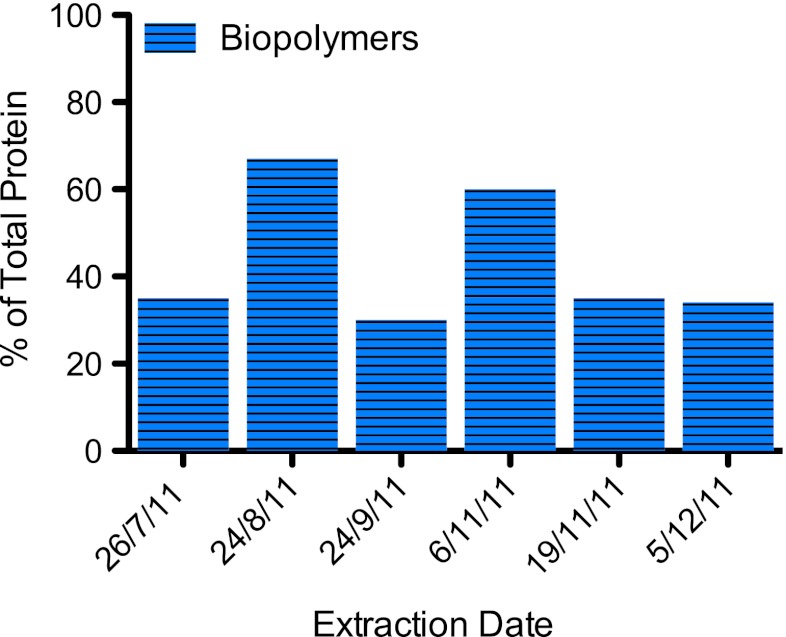
Comparison of biopolymer fractions to total protein from periphyton EPS. Total protein measured was converted to milligrams of carbon to compare with the biopolymer fraction and biopolymer fraction presented as a percentage of total protein as determined by the Bradford method

Polysaccharide and protein were present in similar amounts in EPS extracts and, in most cases, their summation accounted for the measured DOC (Fig. 4). In the August extract, the sum of protein and polysaccharide was greater than measured DOC. Polysaccharide fluctuated between 0.3 and 1.3 mg C/g dry mass and protein between 0.7 and 2.5 mg C/g dry mass over the course of study. Additionally, total protein quantified was compared with the biopolymer fraction (Fig. 5). Calculations of C/N ratios for the low *M*_r_ acid and neutrals fractions were not possible due to the interference of the NO_3_ signal, and therefore these fractions were not included in the comparison with total protein. The biopolymer fraction ranged from 30 to 67 % of the total quantified protein.

## Discussion

### Extraction technique

As the focus of this work was to obtain an EPS extraction technique suitable for use in future metal-binding studies, a protocol was developed to extract DOC from EPS for subsequent metal analysis. Because introducing agitation, such as shaking and stirring, as well as longer periods of extraction time, did not increase the extraction efficiency of DOC (Fig. 1), such steps were not included in the extraction protocol. No significant differences in the types of extracted OC compounds were observed between NaNO_3_ and NaHCO_3_ extraction solutions (Online resource 3 in the ESM), and, therefore, NaNO_3_ was chosen to avoid possible formation of metal-carbonate complexes.

It is important to note that the relative amounts of extracted compounds are dependent upon the method of extraction (Liu and Fang 2002; Nielsen and Jahn 1999; Takahashi et al. 2009). Extraction procedures can be physical methods, as used in this study, or chemical methods used to isolate specific types of compounds, whereby either easily extractable soluble compounds, or more tightly bound compounds are extracted, respectively. Soluble compounds include any soluble macromolecules or colloids, whereas the fraction more strongly bound to cell walls of embedded organisms or to other organic material is considered to contain the less easily extracted compounds of the EPS (Nielsen and Jahn 1999). Characterization of the soluble compounds is more commonly reported in the literature; however, there have also been studies that characterized the more tightly bound fraction (Aguilera et al. 2008; Liang et al. 2010; Sheng et al. 2010). In this study, extracted DOC only reflects OC from soluble and loosely bound EPS. The extraction technique established in this work is suitable when metal speciation is an important consideration. Using sequential chemical extraction techniques can influence properties of the EPS, which can impact metal speciation and produce artifacts (van Hullebusch and Zandvoort 2003). Additionally, using cation exchange resins or EDTA removes metal cations from the EPS (Liang et al. 2010), allowing for the extraction of tightly bound compounds, but also making it difficult to identify specific EPS fractions involved in metal binding.

To assess differences in EPS composition between extracts taken in this study, it was important to determine the variability associated with replicate extractions, which takes into account both the variability associated with biological heterogeneity of the biofilms, as well as that associated with the extraction procedure. Comparison of replicates from extractions performed on two separate occasions showed that the variability is low for DOC and OC compounds over the course of study (Online resource 6 in the ESM). Percent differences between replicates taken in July was less than 10 % for DOC, building blocks, low *M*_r_ acids, and neutrals and less than 20 % for biopolymers. In November, percent differences for DOC and biopolymers was less than 5 % and less than 20 % for the remaining compounds. This analysis shows that differences between replicate extracts is low enough to compare differences between extracts over the period of study.

### EPS composition

Biopolymers, building blocks of HS, low *M*_r_ acids, and neutral/amphiphilic compounds were identified in all EPS extracts and should only reflect either those compounds excreted from organisms, incorporated from the surrounding water, or released intracellular compounds coming from dying or dead organisms. The extraction procedure itself should not have caused the release of additional compounds, as no detectable amount of cell lysis occurred as a result of extraction (Online resource 4 in the ESM). To date, the majority of work analyzing *M*_r_ fractions of EPS comes from bacterial biofilms and there is little work done with EPS from phototrophic biofilms. However, as periphyton are not just comprised of algae, but also contain bacterial communities, the results obtained in this study were compared with the available work conducted with bacterial EPS.

The biopolymer fraction can correspond to high *M*_r_ polysaccharides, which are hydrophilic and non-UV absorbing. Al-Halbouni et al. (2008) reported the biopolymer fraction of EPS taken from a membrane bioreactor (MBR) to be mainly composed of polysaccharides and Hong et al. (2012) reported <1 % of protein in the biopolymer fraction. However, this fraction can also contain amino sugars and proteins. In the present study, the biopolymer fraction was UV absorbing and the C/N ratio was more similar to ratios calculated for protein calibration standards $\left( {\mathrm{C}/\mathrm{N}=0.8-1.1} \right)$ than for PEG standards $\left( {\mathrm{C}/\mathrm{N}=30-40} \right)$. Therefore, as the C/N ratio is influenced by the relative amounts of proteins and polysaccharides, it seems that the biopolymer fraction was largely composed of proteins. In another MBR study, Jiang et al. (2010) also indicated the presence of proteins in the biopolymer peak of soluble microbial products measured in sludge water $\left( {\mathrm{C}/\mathrm{N}=17-18} \right)$, although less than what was measured in the present study.

The biopolymer fraction contained a range of different *M*_r_ proteins, as can be seen by the broad peaks corresponding to this fraction (Fig. 2a). Part of the biopolymer peak contained proteins corresponding to the column separation range (Table 1), while many of these compounds fell within the void volume of the column, corresponding to *M*_r_ larger than 80 kDa. Resolution of individual proteins within the biopolymer fraction having similar *M*_r_ close to the void volume (i.e., BSA and Ovalbumin) was not possible using the OC signal (Online resource 2 in the ESM). Therefore, a column with a higher separation range should be used with LC-OCD-OND for further characterization of the biopolymer fraction.

Studies assessing the *M*_r_ distribution of EPS from microbial biofilms have also reported high *M*_r_ fractions in similar ranges as found in this study. Simon et al. (2009) observed a fraction corresponding to 32–126 kDa in protein equivalent for anaerobic sludge. Another study investigating the *M*_r_ distribution of EPS from activated sludge flocs found two high *M*_r_ fractions corresponding to 16–190 and 270–275 kDa (Comte et al. 2007). Alasonati and Slaveykova (2011) reported specific proteins ranging from 29 to 90 kDa that were identified in EPS from the bacterium *Sinorhizobium meliloti* and, by using asymmetrical flow field-flow fractionation, concluded that a 140-kDa fraction was predominately protein-like substances.

In most extracts, the biopolymer fraction consistently accounted for 30–35 % of the total protein measured using the Bradford assay. This suggests that lower *M*_r_ proteins present in the other LC-OCD-OND fractions may contribute to 65–70 % of the total measured protein. It has been observed that peptides down to 3 kDa are detectable using the Bradford assay (Kruger 2002), and therefore, peptides in the low *M*_r_ acid fraction should be detected. Assays of individual fractions would be needed to determine protein content in the low *M*_r_ acid fraction, as calculation of C/N ratios was not possible because of interference of the NO_3_ signal.

No HS were measured in the EPS extracts, despite being continually measured in the Chriesbach stream water over the course of this 6-month study (data not shown). Liu and Fang (2002) reported humic acids to represent 8.4–30.6 % of extracted EPS from different sludge types, whereas other studies investigating EPS from sludge and wastewater treatment sources cited lower values of 5 and 6.9 % (Frølund et al. 1996; Nielsen and Jahn 1997). A study conducted with periphyton taken from an acidic river showed humic acids to only compose up to 4 % of EPS (Aguilera et al. 2008). The absence of HS in this study is probably because EPS were extracted from newly colonized biofilms after 25 days of growth, and that no HS were incorporated into the EPS from the stream water used to colonize the biofilms. However, building blocks of HS were measured. This fraction, defined as lower *M*_r_ break down products of HS (Huber et al. 2011), was most likely not HS degradation products, but rather small organics incorporated from river water or exudates from organisms. Contrary to what was observed in the biopolymer fraction, the more consistent amounts quantified in extracts (Fig. 3) indicate that the compounds within the building block fraction reflected less the changes taking place within the periphyton community over the course of the 6-month study. The percent of building blocks relative to DOC corresponds well with values found by Hong et al. (2012) who reported values between 11.2 and 19.3 % DOC of foulants in a MBR.

The low *M*_r_ acid peak, corresponding to final degradation products of organics and products from algal and bacterial excretion, is the sum of all free mono- and diprotic low *M*_r_ organic acids. As in the case of building blocks, a narrow *M*_r_ range for the low *M*_r_ acids was observed and did not display significant changes between July and December (Table 1). In agreement with this finding, Comte et al. (2007) reported that a significant portion of low *M*_r_ compounds in EPS extracted from activated sludge were between 0.7 and 2.7 kDa. Hong et al. (2012) did not observe low *M*_r_ acids; however, Al-Halbouni et al. (2008) presented LC-OCD-OND chromatograms with similar percentages of low *M*_r_ acid fractions as found in this study.

The low *M*_r_ neutral/amphiphilic fraction corresponds to alcohols, aldehydes, ketones and amino acids. This fraction measured in our study was in a similar range as found by Hong et al. (2012), who reported slightly lower amounts (25.1–38.3 % DOC). Interestingly, another group reported that the neutral fraction was only observed in the tightly bound EPS that had been extracted using Dowex cation exchange resin and was not present when only centrifugation was used for extraction (Al-Halbouni et al. 2008).

### Applications of LC-OCD-OND in EPS studies

Information on the C/N ratio of the biopolymer fraction could be useful in studying changes in EPS as a result of shifts in species composition from seasonal succession, different stages of biofilm development, or changes in environmental conditions. Changes in extracellular monosaccharide composition of phototrophic biofilms were observed with seasonal changes (D’Souza et al. 2005) and with shifts in community structure (Congestri et al. 2006). A decrease in the total C/N ratio, indicating an increase in protein, of EPS extracted from marine biofilms was observed as a function of biofilm age (D’Souza et al. 2005), and specific proteins were identified necessary for bacterial biofilm formation and development (Southey-Pillig et al. 2005). To monitor such changes, LC-OCD-OND could be used as a comprehensive approach that provides more information than total C/N ratios and is not limited to the analysis of specific saccharides and proteins, as done using HPLC.

The proteinaceous nature of the biopolymer fraction is also of particular interest with respect to metal binding. A study conducted by Guibaud et al. (2003) showed that proteins were an important contributor to the number of binding sites and overall complexation constants that were determined for Cd, Cu, and Pb. Additionally, it was reported that certain *M*_r_ proteins in EPS extracted from periphyton were involved in the binding of Hg (Zhang et al. 2010) and Cu (Zhang and Lee 2012). The metal binding ability of EPS and its role in metal toxicity to periphyton is important to investigate, as many studies have measured total metal accumulation and toxicity to periphyton (Ancion et al. 2010; Bradac et al. 2009a, b; Le Faucheur et al. 2005), but less work has been done to understand how metal binding properties of EPS influence metal bioavailability to periphytic organisms. When coupled to metal analysis, LC-OCD-OND could aid in understanding which components of EPS are significant in metal binding.

## Conclusions

The technique LC-OCD-OND is advantageous over other size based fractionation methods as it quantifies both OC and ON, thus providing additional information about relative amounts of proteins and polysaccharides in different fractions. We have developed both polysaccharide and protein calibrations for better *M*_r_ determination in LC-OCD-OND and this is the first study to use this technique for analysis of EPS originating from periphyton. We have shown that seasonal variation in EPS composition can be detected and quantified, and that LC-OCD-OND has applications beyond purely chemical characterization. Suggested applications include studies of biofilm community structure and metal-EPS binding. Therefore, we conclude that LC-OCD-OND can be used as a new tool for periphyton EPS characterization to simultaneously identify and quantify OC fractions with respect to protein and polysaccharide content as well as *M*_r_ distribution.

## Electronic supplementary material

Below is the link to the electronic supplementary material.

ESM 1 (PDF 598 kb)
