# Supplementary material for: Characterization of extracellular polymeric substances (EPS) from periphyton using liquid chromatography-organic carbon detection–organic nitrogen detection (LC-OCD-OND)
Source: Environ Sci Pollut Res Int. 2012 Oct 12;20(5):3214–23. doi: 10.1007/s11356-012-1228-y (PMC3633784; doi:10.1007/s11356-012-1228-y)
Supplement: Supplementary file 1 — (PDF 598 kb) [file 11356_2012_1228_MOESM1_ESM.pdf]

Characterization of extracellular polymeric substances (EPS) from periphyton using liquid chromatography-organic carbon detection – organic nitrogen detection (LC-OCD-OND)

Environmental Science and Pollution Research

Theodora J. Stewart,[†,‡] Jacqueline Traber,[†] Alexandra Kroll,[†] Renata Behra[†] and Laura Sigg[*†,‡]

[†]*Eawag, Swiss Federal Institute of Aquatic Science and Technology, 8600 Dübendorf, Switzerland*

[‡]*ETH, Institute of Biogeochemistry and Pollutant Dynamics IBP, 8092 Zurich, Switzerland*

*Tel. +41 58 765 54 94; Fax: +41 58 765 53 11; E-mail: laura.sigg@eawag.ch

Electronic Supplementary Material

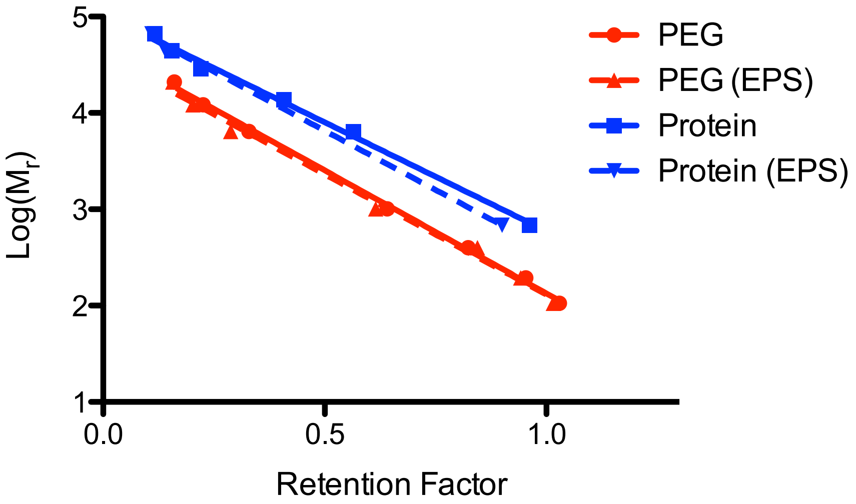

Online Resource 1 LC-OCD-OND calibration curve for polysaccharides and proteins. Polyethyleneglycol (PEG) standards (0.106-21.03 kDa) were diluted with nanopure water (Ω 18, Milli-Q) (—) or spiked into EPS extract (---). Protein standards were diluted with phosphate buffer (20 mM, pH 6.6) and NaCl (150 mM) (—) or spiked into EPS extract (---)

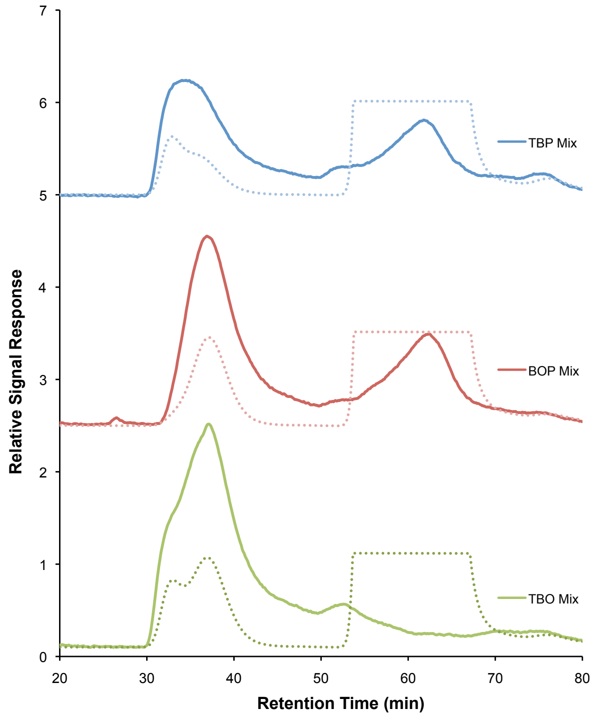

Online Resource 2 Thyroglobulin (T), BSA (B), Ovalbumin (O) and Pepstatin A (P) protein calibration standards combined in mixtures (TBP, BOP, and TBO) in 10 mM $NaNO_3$ and analyzed with LC-OCD-OND. Solid lines represent OC signal and dashed lines represent ON signal. Cutoff of ON signal resulted from high NO_3 concentrations in solution

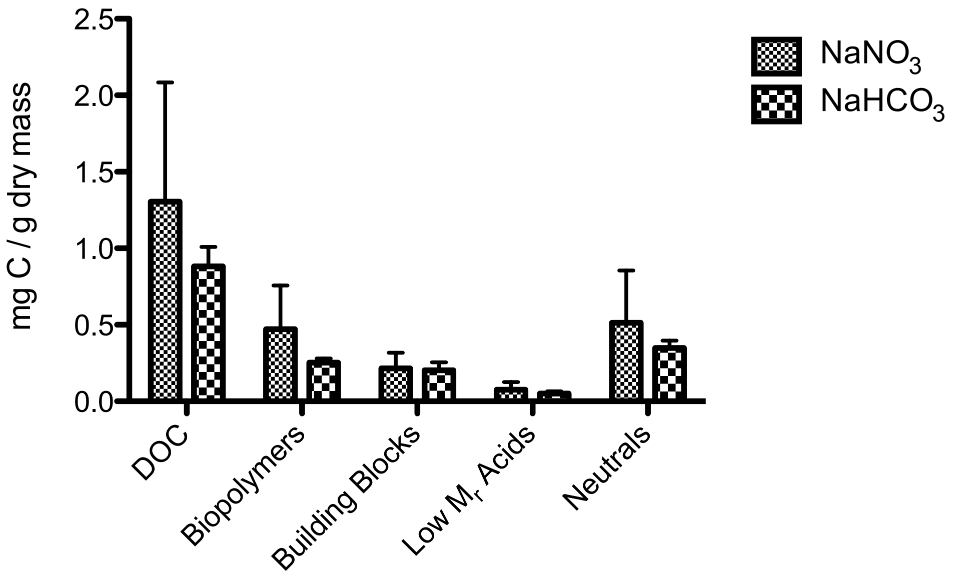

Online Resource 3 Comparison of extraction solutions on OC extraction. Biofilms were harvested and split for extractions in $NaNO_3$ (10 mM, pH 7.4) and $NaHCO_3$ (2mM, pH 7.6). Extracts were analyzed with LC-OCD-OND and OC fractions were quantified and normalized to dry biomass obtained from lyophilization

Online Resource 4 G6P-DH assay results from extraction steps. Lysis of whole biomass with and without protease inhibitors (PI) was used as a representation of 100% of biomass activity

**Sample**	**Activity**	**% Total Activity**
	U/mL	*%*
Cell lysis of biomass (− PI)	0.051 ± 0.002	100
Cell lysis of biomass (+ PI)	0.083 ± 0.002	100
Scraping	Below LOD	0
First sonication		0
Second sonication		0
Centrifugation		0
0.22 μm filtration		0

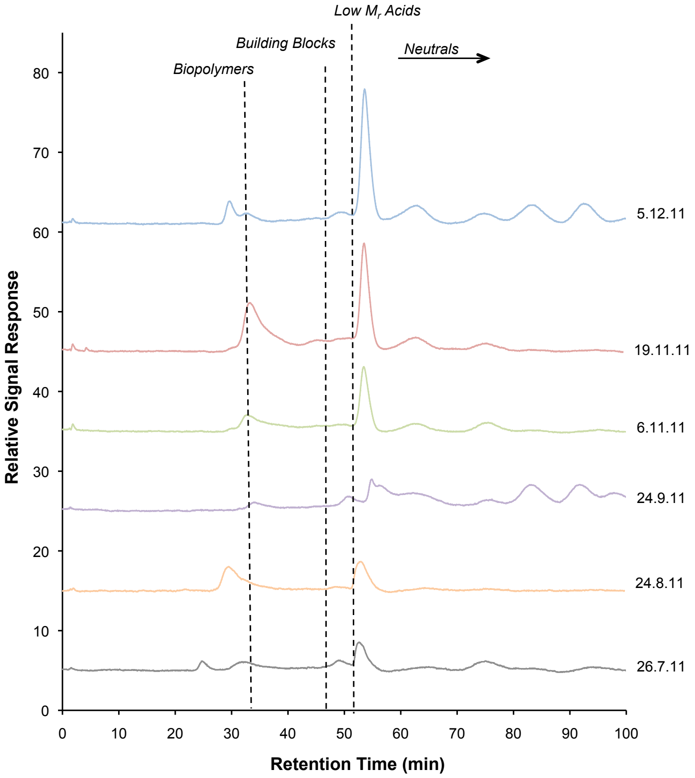

Online Resource 5 UV absorbance of EPS extracts taken between July and December 2011. UV absorbance was detected at 254 nm of OC fractions separated using LC-OCD-OND. Dotted lines represent elution times of corresponding OC fractions.

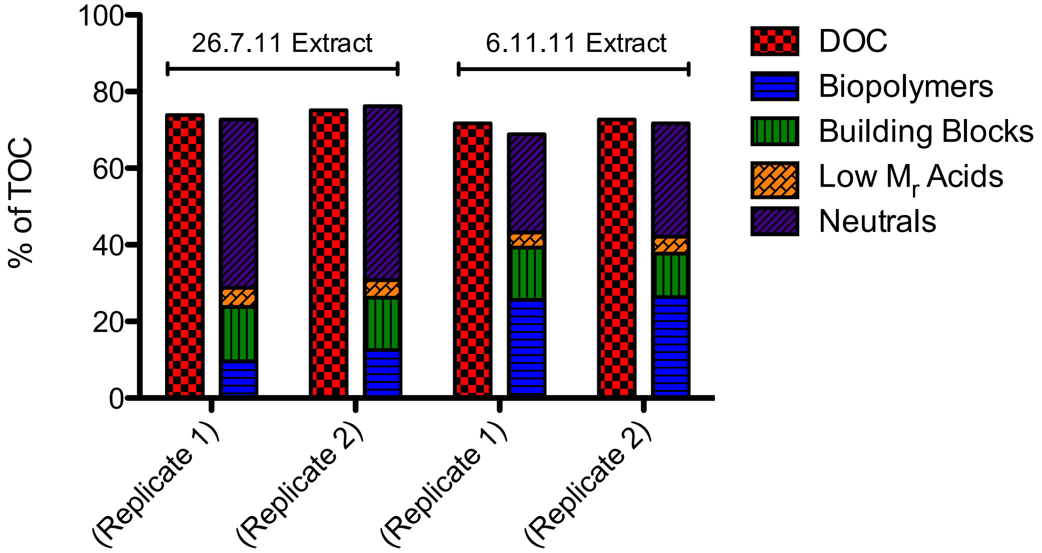

Online Resource 6 Variability of EPS composition. Two separate occasions are displayed when biofilms were collected from one channel and slides split into two groups (replicates 1 and 2). EPS was extracted according to the established protocol for each replicate and extracts were analyzed using LC-OCD-OND. OC fractions were quantified and expressed as % TOC
